# MHD boundary layer radiative, heat generating and chemical reacting flow past a wedge moving in a nanofluid

**DOI:** 10.1186/s40580-014-0020-8

**Published:** 2014-07-01

**Authors:** Md Shakhaoath Khan, Ifsana Karim, Md Sirajul Islam, Mohammad Wahiduzzaman

**Affiliations:** 1 Department of Chemical Engineering, School of Engineering, University of Newcastle, Callaghan, NSW 2308 Australia; 2 Department of Mathematics, Bangabandhu Sheikh Mujibur Rahman Science & Technology University, Gopalganj, 8100 Bangladesh; 3 Mathematics Discipline, Science Engineering and Technology School, Khulna University, Khulna, 9208 Bangladesh

**Keywords:** Nanofluid dynamics, Wedge, Magnetic field, Thermal radiation, Heat generation, Chemical reaction

## Abstract

The present study analyzed numerically magneto-hydrodynamics (MHD) laminar boundary layer flow past a wedge with the influence of thermal radiation, heat generation and chemical reaction. This model used for the momentum, temperature and concentration fields. The principal governing equations is based on the velocity *u*_*w*_(x) in a nanofluid and with a parallel free stream velocity *u*_*e*_(x) and surface temperature and concentration. Similarity transformations are used to transform the governing nonlinear boundary layer equations for momentum, thermal energy and concentration to a system of nonlinear ordinary coupled differential equations with fitting boundary conditions. The transmuted model is shown to be controlled by a number of thermo-physical parameters, viz. the magnetic parameter, thermal convective parameter, mass convective parameter, radiation-conduction parameter, heat generation parameter, Prandtl number, Lewis number, Brownian motion parameter, thermophoresis parameter, chemical reaction parameter and pressure gradient parameter. Numerical elucidations are obtained with the legendary Nactsheim-Swigert shooting technique together with Runge–Kutta six order iteration schemes. Comparisons with previously published work are accomplished and proven an excellent agreement.

## 1 Background

Falkner and Skan [1] were firstly established a viscous fluid flow in excess of a static wedge by employing similarity transformation that can be utilized to reduce the limited differential boundary layer equations to a nonlinear third-order normal differential equation. In the past few years researchers shows interest on Falkner-Skan flow considering various parameter effects. Hartree [2] investigated the explanations and dependence on *β*. Koh and Hartnett [3] predicted the skin-friction and heat transfer for the boundary layer flow over porous wedges. Also the steady two dimensional laminar heat transfer flow from a wedge was measured by Lin and Lin [4].

Watanabe [5] investigated thermal boundary layer flow over a uniform surface temperature wedge with a transpiration velocity in forced flow. Khan et al. [6] have studied the unsteady mixed convective boundary layer flow from a vertical porous plate with induced magnetic field and heat generation. Hossain et al. [7] studied also the problem by having temperature dependent viscosity as well as thermal conductivity on the forced flow past a wedge and heat transfer of a viscous incompressible fluid with uniform surface heat flux. Recently, there have been relatively few analysis [8-11] described the boundary layer equations of the laminar flow within wedge with different angles from flat plate at zero incidences to two dimensional stagnation flows. Postelnicu and Pop [12] analyzed the stretching wedge problem of Falkner-Skan boundary layer flow of a power-law fluid.

It is now well accepted fact that the terms magnetohydrodynamics (MHD), thermal radiation and heat generation extensively appear in various engineering processes. MHD is significant in the control of boundary layer flow and metallurgical processes. Again the thermal radiation and heat generation possessions may arise in high temperature ingredients processing operations. Ingredients may be intelligently designed therefore with judicious implementation of radiative heating to produce the desired characteristics. This recurrently occurs in agriculture, engineering, plasma studies and petroleum industries.

Numerous flow complications under different aspects have been considered by the several scholars. Vajravelu and Hadjinicalaou [13] scrutinized the heat transfer characteristics over a stretching surface with viscous dissipation in the presence of internal heat generation or absorption. The effect of radiation on convective heat transfer problems have been examined by a number of researchers using principally algebraic approximations for the radiative transfer simulation. Takhar *et al.* [14] employed a differential non-gray gas approximation to study nonlinear gas dynamics in a permeable material. Seddeek [15] evaluated the effects of radiation and variable viscosity on hydromagnetic convection flow with an aligned magnetic field using a numerical method and a flux approximation for radiation. Bég *et al.* [16] used a Rosseland diffusion flux model to investigate transient radiative-convection boundary layer flow in porous media with an electrical network simulator.

In recent years studies on nanofluid heat and mass transfer boundary layer laminar flow have attracted considerable attention. Nanotechnology [17] has been broadly used in several industrial applications. Nanofluids demonstrate anomalously high thermal conductivity, significant change in properties such as viscosity and specific heat in comparison to the base fluid, features which have attracted many researchers to perform in engineering applications. Convective instability and heat transfer characteristics of nanofluids were studied by Kang and Choi [18]. Kang *et al.* [19] experimentally investigated on nanofluids include thermal conductivity. Jang and Choi [20] reconnoitered nanofluid thermal conductivity parameter effects. Nield and Kuznestov [21] and Kuznestov and Nield [22] considered laminar convective nanofluid boundary layer flow in a porous medium, with Brownian motion and thermophoresis particle deposition effects and simple boundary conditions. Khan and Pop [23,24] studied boundary layer heat-mass transfer free convection flows also in porous media of a nanofluid past a stretched sheet. Hamad and Pop [25] reported transient hydro magnetic free convection rotating flow of a nanofluid. Md. Shakhaoath Khan et al. [26] analyzed the boundary layer nanofluid flow with MHD radiative possessions. And Khan and Pop [27] investigates boundary layer heat and mass transfer analysis past a wedge moving in a nanofluid.

The prime objective of the present attempt is to extend the analysis of Khan and Pop [27]. This study finds the effect of thermal radiation, heat generation and chemical reaction on themagneto hydrodynamic convection flow past a wedge moving in a nanofluid. This study also emphasised that Brownian motion and thermophoresis are significant mechanisms in nanofluid performance. This study is encouraged by precise application in materials processing which combines photopyroelectric thermal radiation and magnetic fields simultaneously to modify nanofluid properties. The resulting non-dimensionalized two-point boundary value problem is solved subject to physically realistic boundary conditions with the Nactsheim-Swigert shooting scheme [28]. Verification of computations is demonstrated by comparison with previously published literature of Khan and Pop [27], White [29], Yih [30] and Yacob et al. [31]. The present study is applicable to the manufacturing of magnetic nanofluids and chemical engineering operations involving electro-conductive nano fluid suspensions. There are relatively few studies [32-43] also focused on the MHD, convection, radiative heat transfer, heat generation and nanofluid also addressed application for further research.

## 2 Methods: Mathematical model

**Figure 1:**
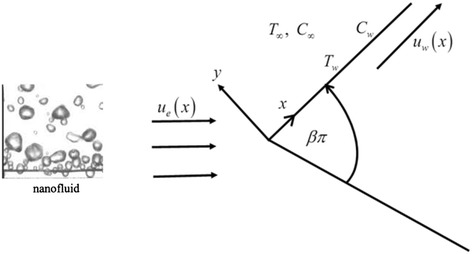
**Physical configuration of a wedge.**

Assuming the two dimensional MHD laminar boundary layer heat and mass transfer flow past an impermeable stretching wedge with the influence of thermal radiation, heat generation and chemical reactionand moving with the velocity *u*_*w*_(*x*) in a nanofluid, and the free stream velocity is *u*_*e*_(*x*), where *x* is the coordinate measured along the surface of the wedge. The sketch of the physical configuration and coordinate system are shown in Figure 1 by following Khan and Pop [27].

Here *u*_*w*_(*x*) >*0* corresponds to a stretching wedge surface velocity and *u*_*w*_(*x*) < 0 corresponds to a contracting wedge surface velocity, respectively. Instantaneously at time *t* > 0, temperature of the plate and species concentration are raised to *T*_*w*_( > *T*_∞_) and *C*_*w*_( > *C*_∞_) respectively, which are thereafter maintained constant, where *T*_*w*_ , *C*_*w*_ are temperature and species concentration at the wall and *T*_∞_, *C*_∞_ are temperature and species concentration far away from the plate respectively. A strong magnetic field *B* = (0, *B*_0_, 0) is applied in the *y-*direction. Under the above assumptions and usual boundary layer approximation, the MHD Mixed convective nanofluid flow governed by the following equations (see [21] and [22]);

1 $$ \frac{\partial u}{\partial x}+\frac{\partial v}{\partial y}=0, $$

2 $$ u\frac{\partial u}{\partial x}+ v\frac{\partial u}{\partial y}={u}_e\frac{du}{dx}+\nu \frac{\partial^2 u}{\partial {y}^2}+ g{\beta}_T\left( T-{T}_{\infty}\right)+ g{\beta}_C\left( C-{C}_{\infty}\right)-\frac{\sigma {B_0}^2 u}{\rho}, $$

3 $$ u\frac{\partial T}{\partial x}+ v\frac{\partial T}{\partial y}=\alpha \frac{\partial^2 T}{\partial {y}^2}+\frac{Q_{\circ }}{\rho {C}_p}\left( T-{T}_{\infty}\right)+\frac{16}{3}\frac{\sigma_1{T}_{\infty}^3}{\rho {C}_p{k}_1}\frac{\partial^2 T}{\partial {y}^2}+\tau \left\{{D}_B\left(\frac{\partial T}{\partial y}\cdot \frac{\partial C}{\partial y}\right)+\frac{D_T}{T_{\infty }}{\left(\frac{\partial T}{\partial y}\right)}^2\right\}, $$

4 $$ u\frac{\partial C}{\partial x}+ v\frac{\partial C}{\partial y}={D}_B\frac{\partial^2 C}{\partial {y}^2}+\frac{D_T}{T_{\infty }}\frac{\partial^2 T}{\partial {y}^2}-{k}_r\left( C-{C}_{\infty}\right). $$

Here in equation (2) the 3rd term on the right hand side is the convection due to thermal expansion and gravitational acceleration, the 4th term on the right hand side is the convection due to mass expansion and gravitational acceleration and the 5th term generated by the magnetic field strength because a strong magnetic field *B* = (0, *B*_0_, 0) is applied in the *y-*direction.

Again in equation (3) the 2nd term on the right hand side is the effect of heat generation [40] on temperature flow, 3rd term on the right hand side expressed the radiative [32] heat transfer flow, and the last term indicates the Brownian motion due to nanofluid heat and mass transfer flow.

Also in equation (3) the 2nd term on the right hand side is the thermophoresis diffusion term due to nanofluid flow and the 3rd term is the rate of chemical reaction on the net mass flows.

And the boundary condition for the model is;

5 $$ u={u}_w(x)=-\lambda {u}_e(x), v=0, T={T}_w, C={C}_w\;\mathrm{at}\; y=0, u={u}_e(x), T\to {T}_{\infty }, C\to {C}_{\infty}\;\mathrm{as}\  y\to \infty . $$

In order to conquers a similarity solution to eqs. (1) to (4) with the boundary conditions (5) the following similarity transformations, dimensionless variables are adopted in the rest of the analysis;

6 $$ \begin{array}{l}\eta = y\left(\sqrt{\frac{\left(1+ m\right){u}_e}{2 x\nu}}\right),\kern0.36em \psi =\sqrt{\left(\frac{2{u}_e x\nu}{1+ m}\right)} f\left(\eta \right),\kern0.36em \theta =\theta \left(\eta \right)=\frac{T-{T}_{\infty }}{T_w-{T}_{\infty }},\\ {}\varphi =\varphi \left(\eta \right)=\frac{C-{C}_{\infty }}{C_w-{C}_{\infty }}\kern0.48em \mathrm{and}\kern0.48em  u=\frac{\partial \psi}{\partial y}, v=-\frac{\partial \psi}{\partial x},\end{array} $$

For the similarity solution of equation (1) to (4) with considering the value (from the properties of wedge, see reference [27]) *u*_*e*_(*x*) = *ax* ^*m*^, *u*_*w*_(*x*) = *cx* ^*m*^, where *a, c* and *m* (0 ≤ *m* ≤ 1) are positive constant. Therefore, the constant moving parameter *λ* is defined as *λ = c/a*, whereas *λ* < 0 relates to a stretching wedge, *λ* > 0 relates to a contracting wedge, and *λ* = 0 corresponds to a fixed wedge, respectively.

From the above transformations the non-dimensional, nonlinear, coupled ordinary differential equations are obtained as;

7 $$ {f}^{///}\left(\eta \right)+ f\left(\eta \right){f}^{//}\left(\eta \right)+\beta \left[1-{f}^{/}{\left(\eta \right)}^2\right]+{\lambda}_T\theta \left(\eta \right)+{\lambda}_M\varphi \left(\eta \right)- M{f}^{/}\left(\eta \right)=0, $$

8 $$ \left(\left( 1+ R\right)/{P}_r\right){\theta}^{//}\left(\eta \right)+ f\left(\eta \right){\theta}^{/}\left(\eta \right)+{N}_b{\theta}^{/}\left(\eta \right){\varphi}^{/}\left(\eta \right)+{N}_t{\theta}^{/}{\left(\eta \right)}^2+ Q\theta \left(\eta \right)=0, $$

9 $$ {\varphi}^{//}\left(\eta \right)+\left({N}_t/{N}_b\right){\theta}^{//}\left(\eta \right)+{L}_e\; f\left(\eta \right){\varphi}^{/}\left(\eta \right)-\gamma {R}_e{L}_e\;\varphi \left(\eta \right)=0. $$

The transformed boundary conditions are as follows;

10 $$ \left.\begin{array}{c}\hfill f= 0,{f}^{/}=-\lambda, \theta = 1,\varphi = 1,\kern1.2em  at\kern0.24em \eta = 0\hfill \\ {}\hfill {f}^{/}= 1,\theta = 0,\varphi = 0,\kern3.12em  as\kern0.24em \eta \to \infty .\hfill \end{array}\right\} $$

where the notation primes denote differentiation with respect to *η* and the parameters are defined as:

Magnetic parameter, $$ M=\frac{\sigma {B_0}^22}{\rho \left( m+1\right){u}_e}, $$

Pressure gradient parameter, $\beta =\frac{2 m}{m+1},$

Grashof number, $$ {G}_r=\frac{2 g{\beta}_T\left({T}_w-{T}_{\infty}\right){x}^3}{\left( m+1\right){\nu}^2}, $$

Modified Grashof number, ${G}_m=\frac{2 g{\beta}_C\left({C}_w-{C}_{\infty}\right){x}^3}{\left( m+1\right){\nu}^2},$

Thermal convective parameter, ${\lambda}_T=\frac{G_r}{{R_e}^2},$

Mass convective parameter, ${\lambda}_M=\frac{G_m}{{R_e}^2},$

Local Reynolds number, ${R}_e=\frac{x{ u}_e}{\nu},$

Prandtl number, $$ {P}_r=\frac{\upsilon}{\alpha}, $$

Heat source parameter, $Q=\frac{2{Q}_{\circ } x}{\rho {C}_p\left( m+1\right){u}_e},$

Lewis number, $$ {L}_e=\frac{\upsilon}{D_B}, $$

Brownian motion parameter, ${N}_b=\frac{{\left(\rho c\right)}_p{D}_B\left({C}_w-{C}_{\infty}\right)}{\upsilon {\left(\rho c\right)}_f},$

Thermophoresis parameter, $$ {N}_t=\frac{{\left(\rho c\right)}_p{D}_T\left({T}_w-{T}_{\infty}\right)}{\upsilon {T}_{\infty }{\left(\rho c\right)}_f}, $$

Radiation parameter, $R=\frac{ 1 6}{3}\frac{\sigma_1{T}_{\infty}^3}{\rho {C}_p{k}_1\nu}$ and

Chemical reaction parameter, $\gamma =\frac{2{ k}_r\nu}{{u_e}^2\left( 1+ m\right)}.$

Significant prominent that numerous of non-dimensionalized thermo-fluid constraints are known as “local parameters”. This methodology is effective and has been in practice for a number of years. It is an effectual methodology which engrosses the x-dependence into correctly scaled dimensionless numbers. The solutions remain valid and correct and the reviewer is referred to the following references corroborating this approach- Khan and Gorla [44] and Mahmood*et al*. [45]. In this context, *M* is a local magnetic body force number (Mahmood*et al*. [45]) and *λ*_*T*_ is therefore a function of *local* thermal Grashof number and *λ*_*m*_ is a function of *local* species Grashof number (Khan and Gorla [44]).

## 3 Numerical (Shooting Quadrature) simulations

The non dimensional, nonlinear, coupled ordinary differential equations (7) to (9) with boundary condition (10) are solved numerically using standard initially value solver the shooting method. For the purpose of this method, the Nactsheim-Swigert shooting iteration technique [28] together with Runge–Kutta six order iteration scheme is taken and determines the temperature and concentration as a function of the coordinate *η*. A method for the numerical solution of the differential equations of the boundary layer type was presented by Philip R. Nachtsheim and Paul Swigert in 1965. For the purpose of present problem, we applied the Nacthsheim-Swigert iteration technique.

In shooting method, the missing (unspecified) initial condition at the initial point of the interval is assumed and the differential equation is integrated numerically as an initial value problem to the terminal point. The accuracy of the assumed missing initial condition is then checked by comparing the calculated value of the dependent variable at the terminal point with its given value there. If a difference exists, another value of the missing initial condition must be assumed and the process is repeated. This process is continued until the agreement between the calculated and the given condition at the terminal point is within the specified degree of accuracy. For this type of iterative approach, one naturally inquires whether or not there is a systematic way of finding each succeeding (assumed) value of the missing initial condition.

The boundary conditions equation (10) associated with the ordinary nonlinear differential equations of the boundary layer type is of the two-point asymptotic class. Two-point boundary conditions have values of the dependent variable specified at two different values of the independent variable. Specification of an asymptotic boundary condition implies the value of velocity approaches to unity and the value of temperature approaches to zero as the outer specified value of the independent variable is approached. The method of numerically integrating two-point asymptotic boundary value problem of the boundary layer type, the initial value method, requires that the problem be recast as an initial value problem. Thus it is necessary to set up as many boundary conditions at the surface as there are at infinity. The governing differential equations are then integrated with these assumed surface boundary conditions. If the required outer boundary condition is satisfied, a solution has been achieved.

However, this is not generally the case. Hence a method must be devised to logically estimate the new surface boundary conditions for the next trial integration. Asymptotic boundary value problems such as those governing the boundary layer equations are further complicated by the fact that the outer boundary condition is specified at infinity. In the trial integration infinity is numerically approximated by some large value of the independent variable. There is no a priori general method of estimating this value. Selection of too small a maximum value for the independent variable may not allow the solution to asymptotically converge to the required accuracy. Selecting a large value may result in divergence of the trial integration or in slow convergence of surface boundary conditions required satisfying the asymptotic outer boundary condition. Selecting too large a value of the independent variable is expensive in terms of computer time. Nachtsheim-Swigert developed an iteration method, which overcomes these difficulties. Extension of the iteration shell to above equation system of differential equations (10) is straightforward, there are three asymptotic boundary condition and hence three unknown surface conditions *f* ^//^(0), *θ* ^/^(0) and *φ* ^/^(0).

## 4 Results and discussion

In order to investigate the physical representation of the problem, the numerical values of velocity (*f* ^/^), temperature (*θ*) and concentration (*φ*) have been computed for resultant principal parameters as the Magnetic parameter *M*, pressure gradient parameter *β*, Thermal convective parameter *λ*_*T*_, Mass convective parameter *λ*_*M*_, local Reynolds number *R*_*e*_, Prandtl number *P*_*r*_, Heat source parameter *Q*, Lewis number *L*_*e*_, Brownian motion parameter *N*_*b*_, thermophoresis parameter *N*_*t*_, radiation parameter *R* and chemical reaction parameter *γ* respectively. To assess the accuracy of the numerical results the Skin friction coefficient *f* ^//^(0) compared with previous literatures. And excellent agreement is observed.

The prediction of Skin friction coefficient *f* ^//^(0) for several values of when *λ* = *λ*_*T*_ = *λ*_*M*_ = *M* = *R* = *Q* = *γ* = 0 have been compared White [29], Yih [30], Yacob et al. [31], and Khan and Pop [27] and shown in Table 1. It is seen from the above table Skin friction coefficient rises progressively which precisely complemented with the previous published data. The physical representation of the present analysis is shown in Figures 2, 3, 4, 5, 6, 7, 8, 9, 10, 11, 12, 13 and 14.

**Table 1:** **Comparison of the values of Skin friction coefficient** *f* ^//^(0) **for several values of** ***m*** **when** *λ* = *λ*_*T*_ = *λ*_*M*_ = *M* = *R* = *Q* = *γ* = 0

***m***	**White** [29]	**Yih** [30]	**Yacob et al.** [31]	**Khan and Pop** [27]	**Present result**
0	0.4696	0.4696	0.4696	0.4696	0.4699
1/11	0.6550	0.6550	0.6550	0.6550	0.6574
1/5	0.8021	0.8021	0.8021	0.8021	0.8045
1/3	0.9277	0.9276	0.9276	0.9277	0.9298
1/2	1.0389	-	-	1.0389	1.0394
1	1.2326		1.2326	1.2326	1.2358

**Figure 2:**
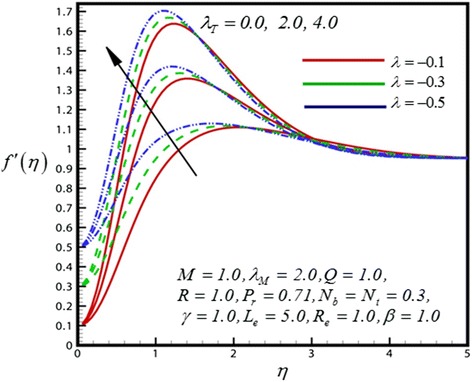
**Velocity profile for different values of** ***λ***_***T***_ **.**

**Figure 3:**
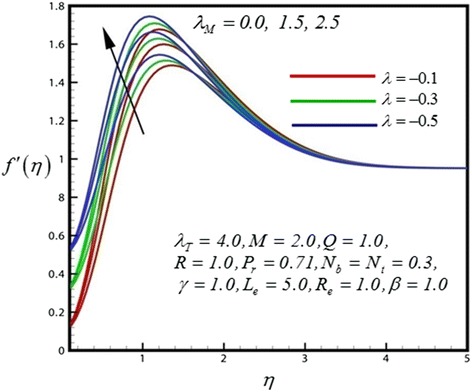
**Velocity profile for different values of** ***λ***_***M***_ **.**

**Figure 4:**
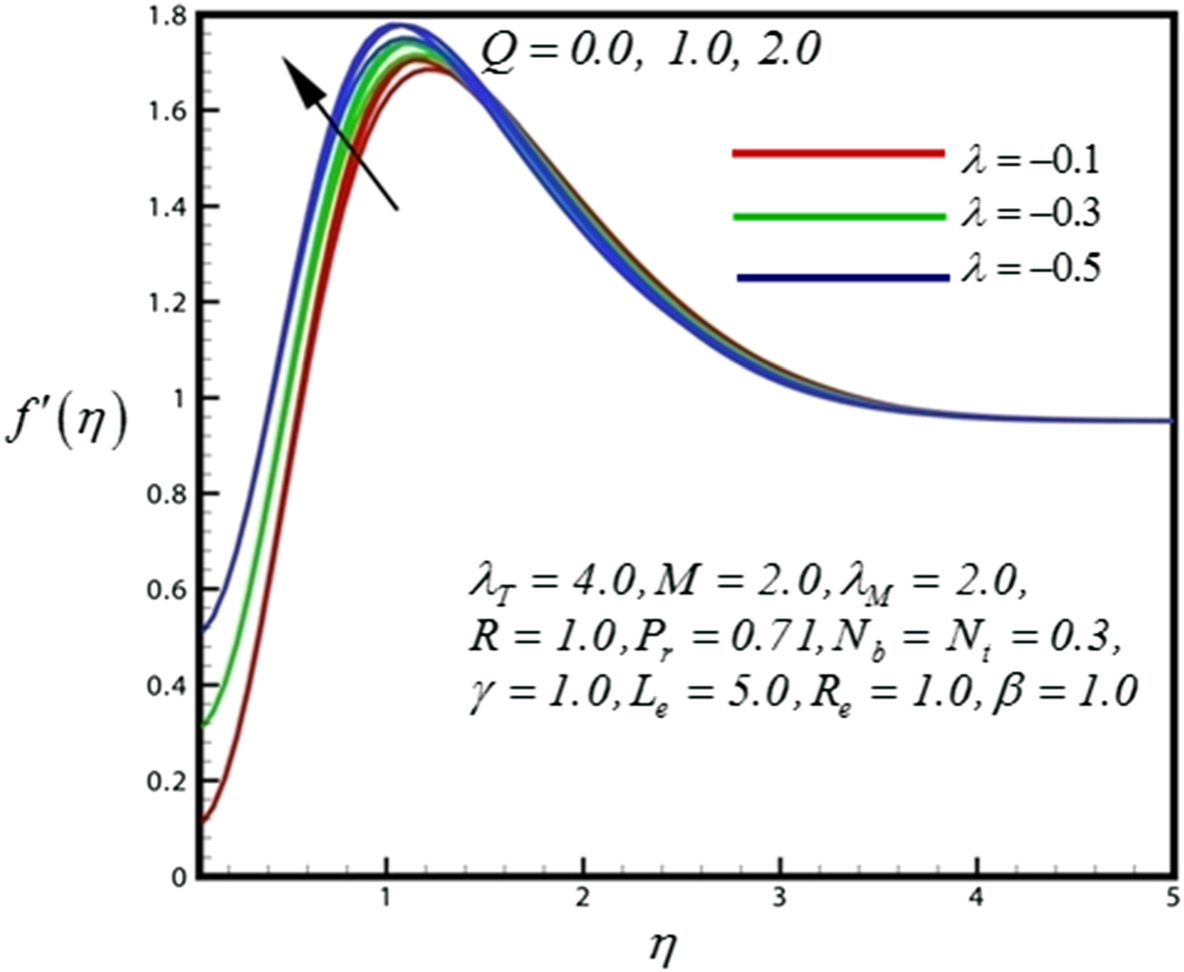
**Velocity profile for different values of** ***Q*** **.**

**1:**
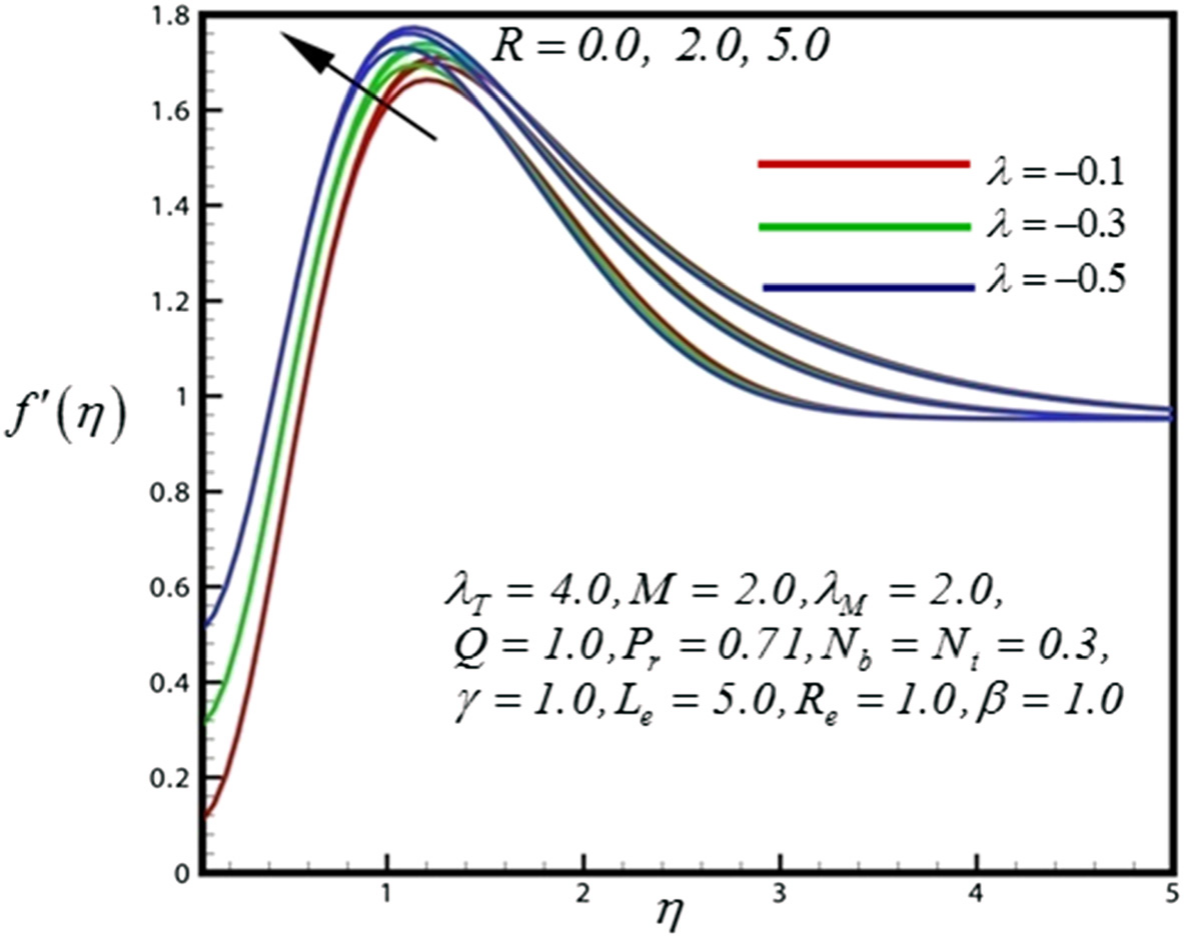
**Velocity profile for different values of** ***R*** **.**

**Figure 6:**
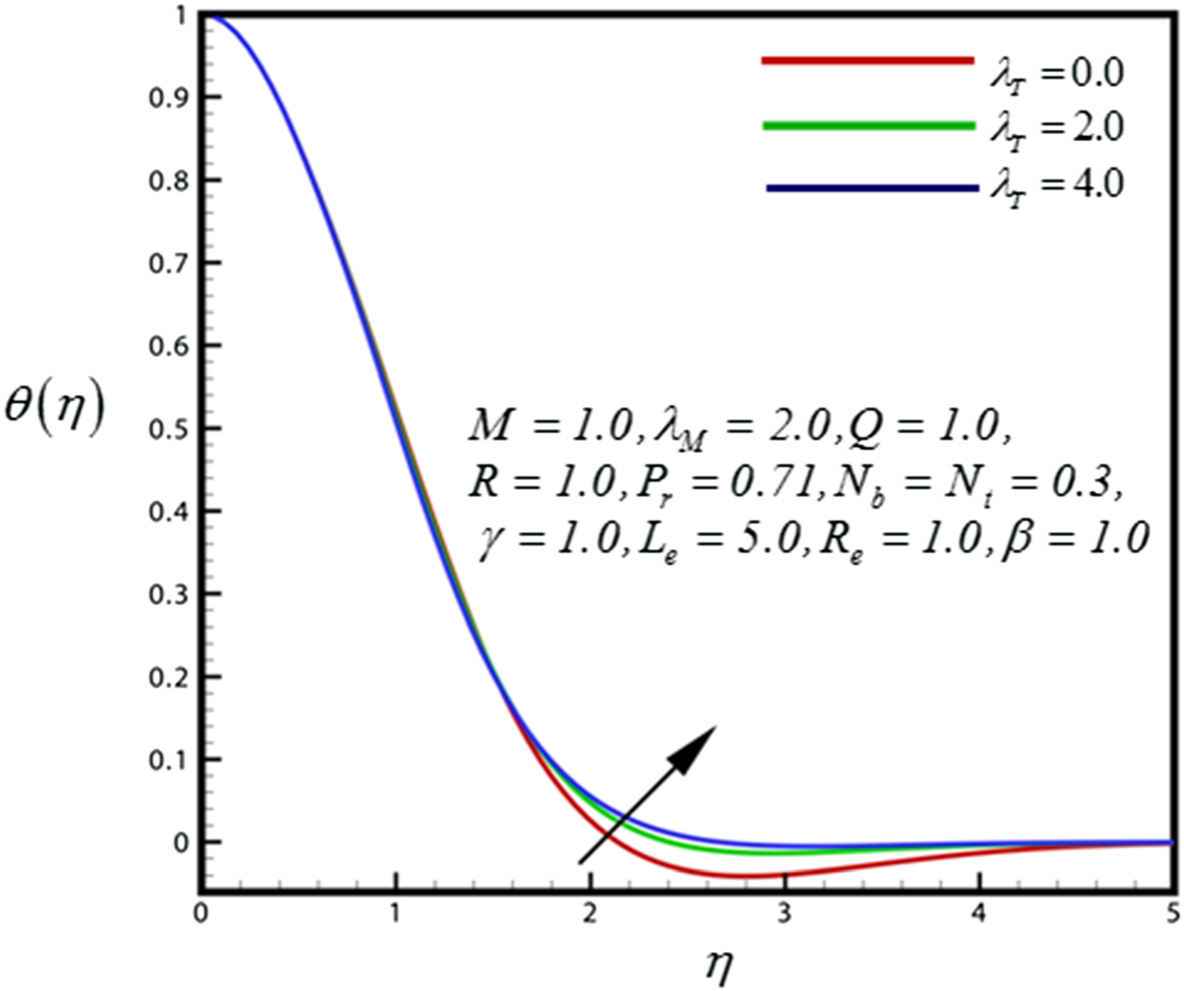
**Temperature profile for different values of** ***λ***_***T***_ **.**

**Figure 7:**
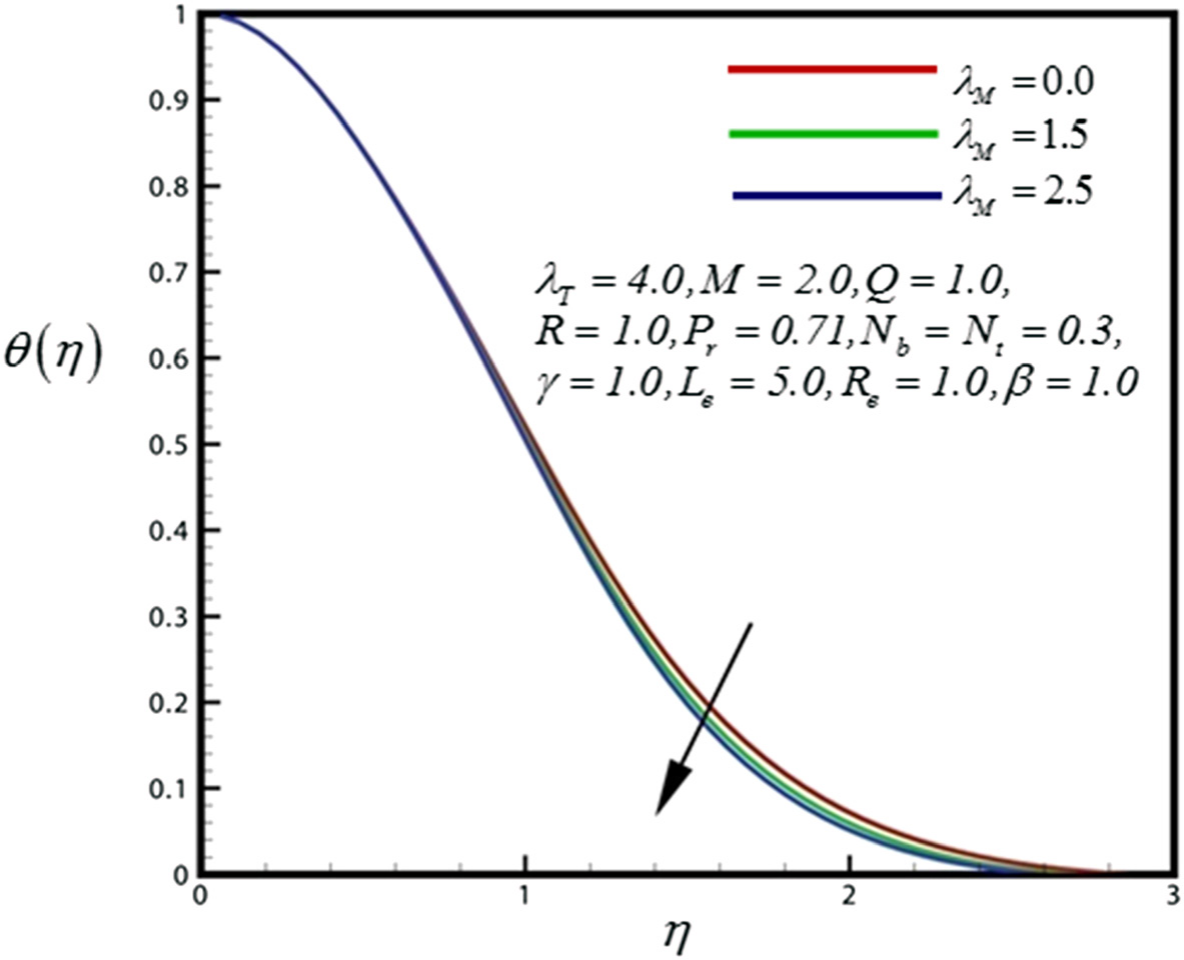
**Temperature profile for different values of** ***λ***_***M***_ **.**

**Figure 8:**
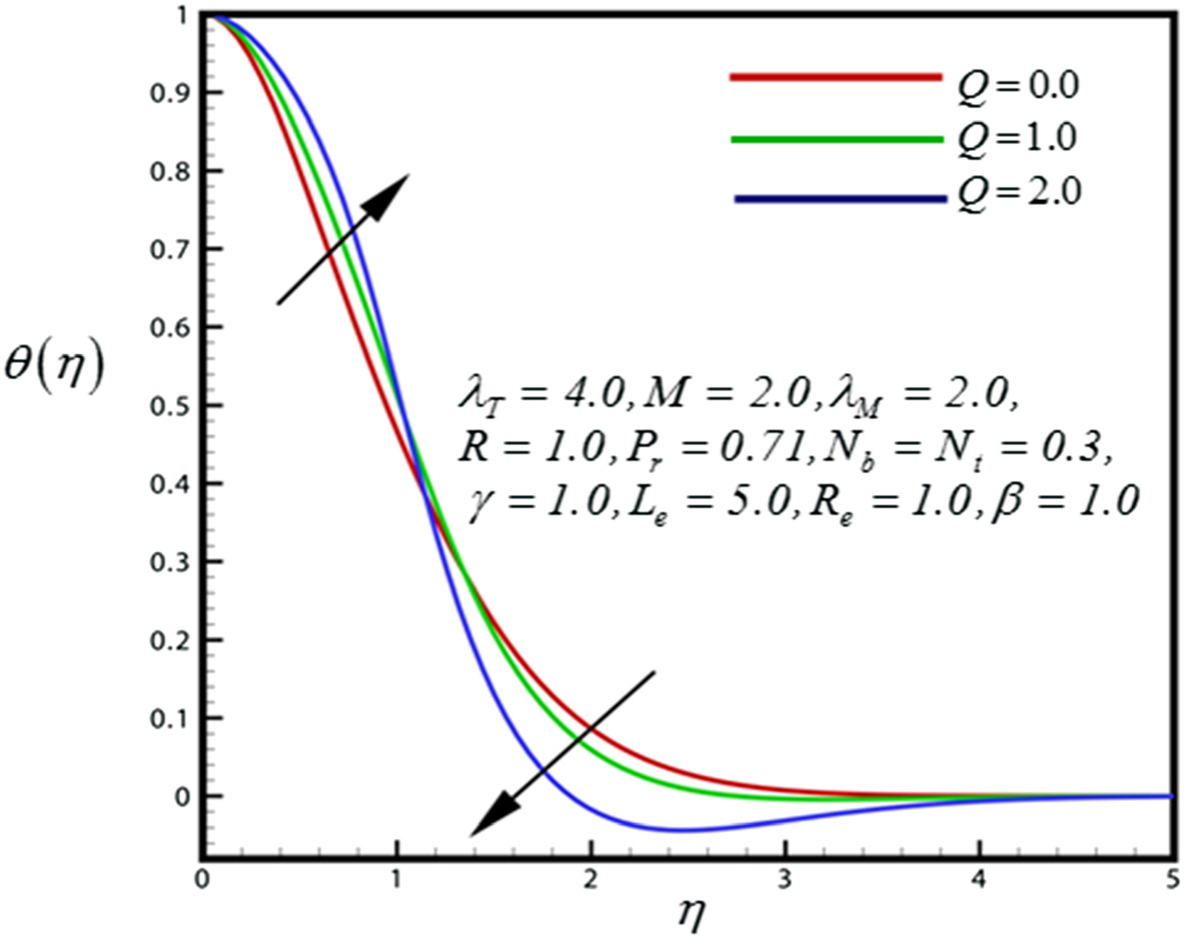
**Temperature profile for different values of** ***Q*** **.**

**Figure 9:**
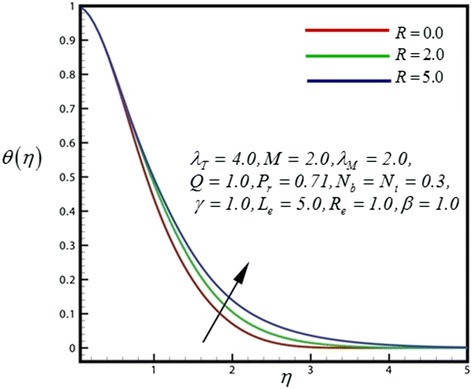
**Temperature profile for different values of** ***R*** **.**

**Figure 10:**
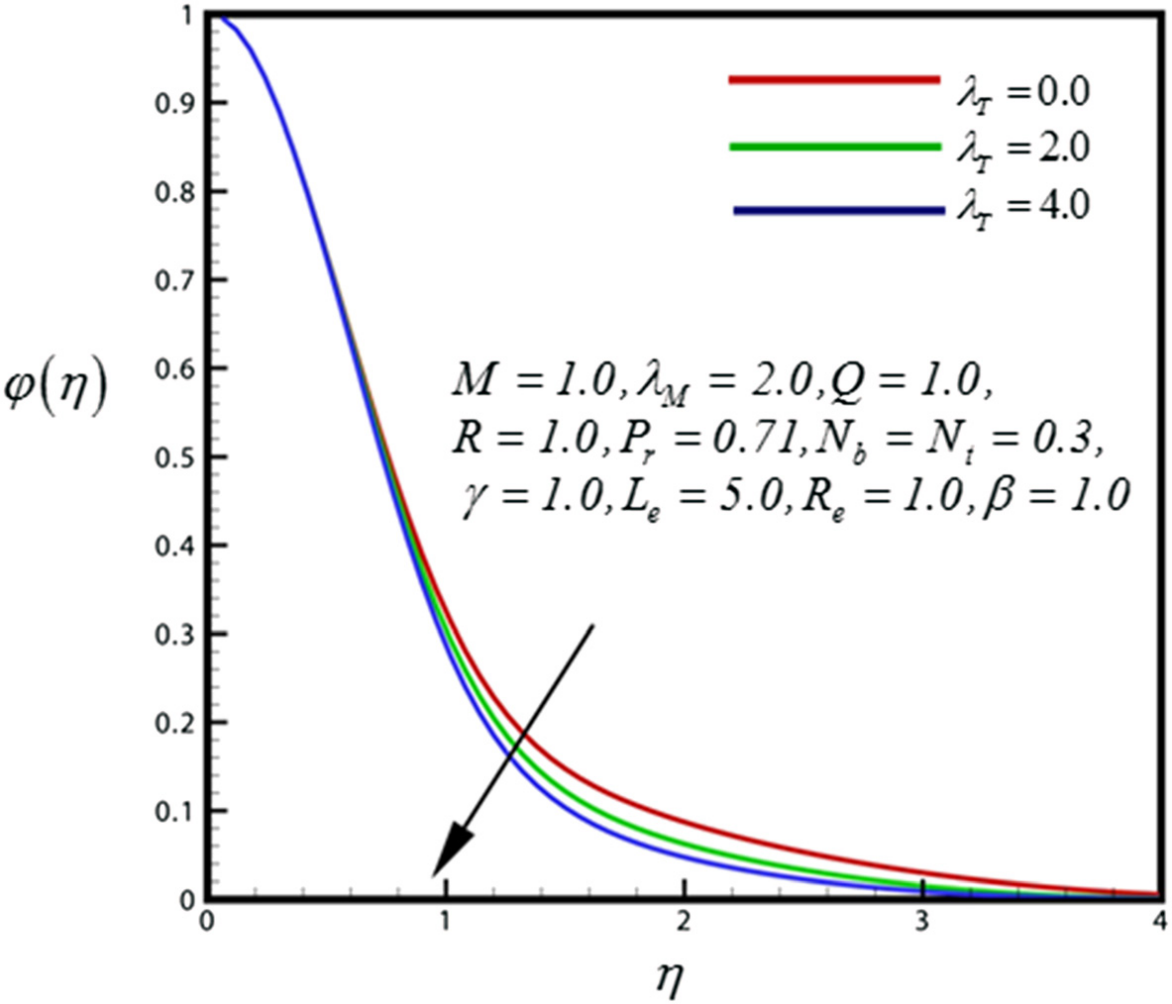
**Concentration profile for different values of** ***λ***_***T***_ **.**

**Figure 11:**
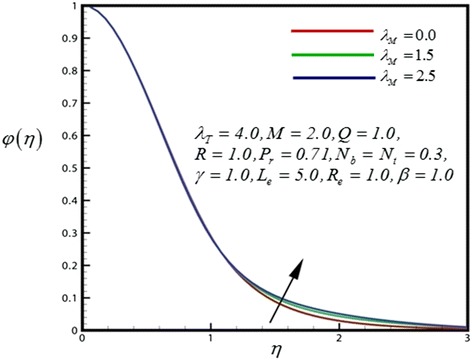
**Concentration profile for different values of** ***λ***_***M***_ **.**

**Figure 12:**
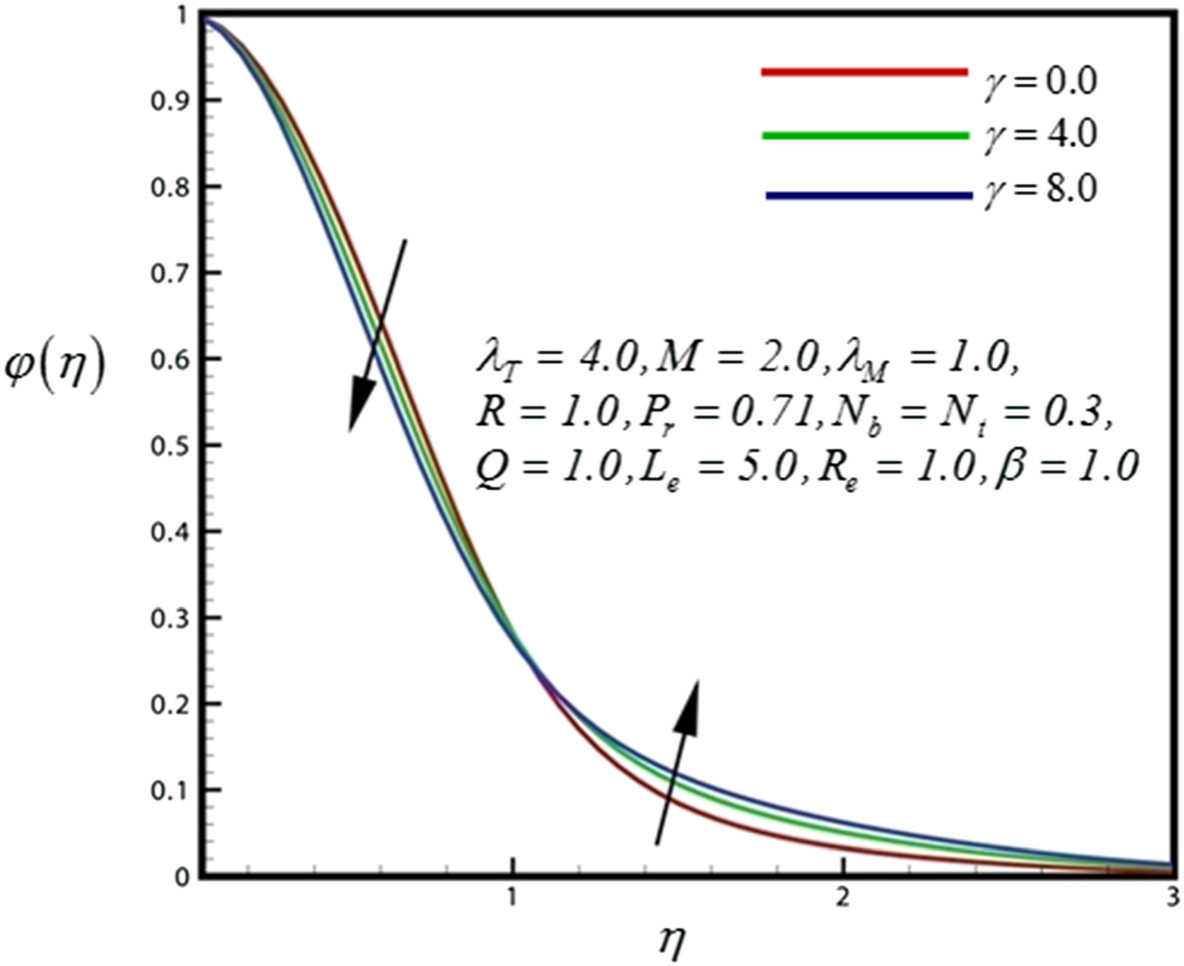
**Concentration profile for different values of** ***γ*** **.**

**Figure 13:**
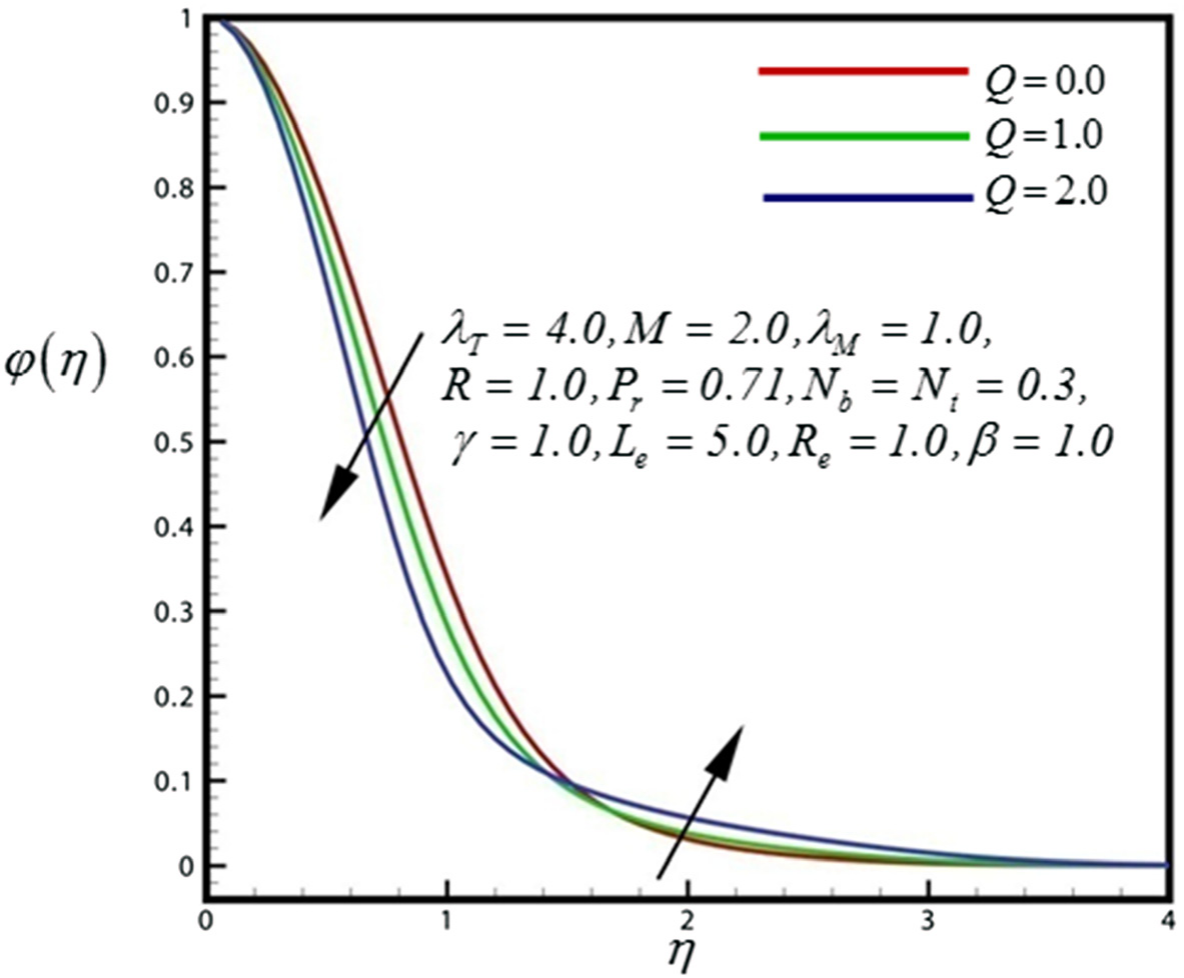
**Concentration profile for different values of** ***Q*** **.**

**Figure 14:**
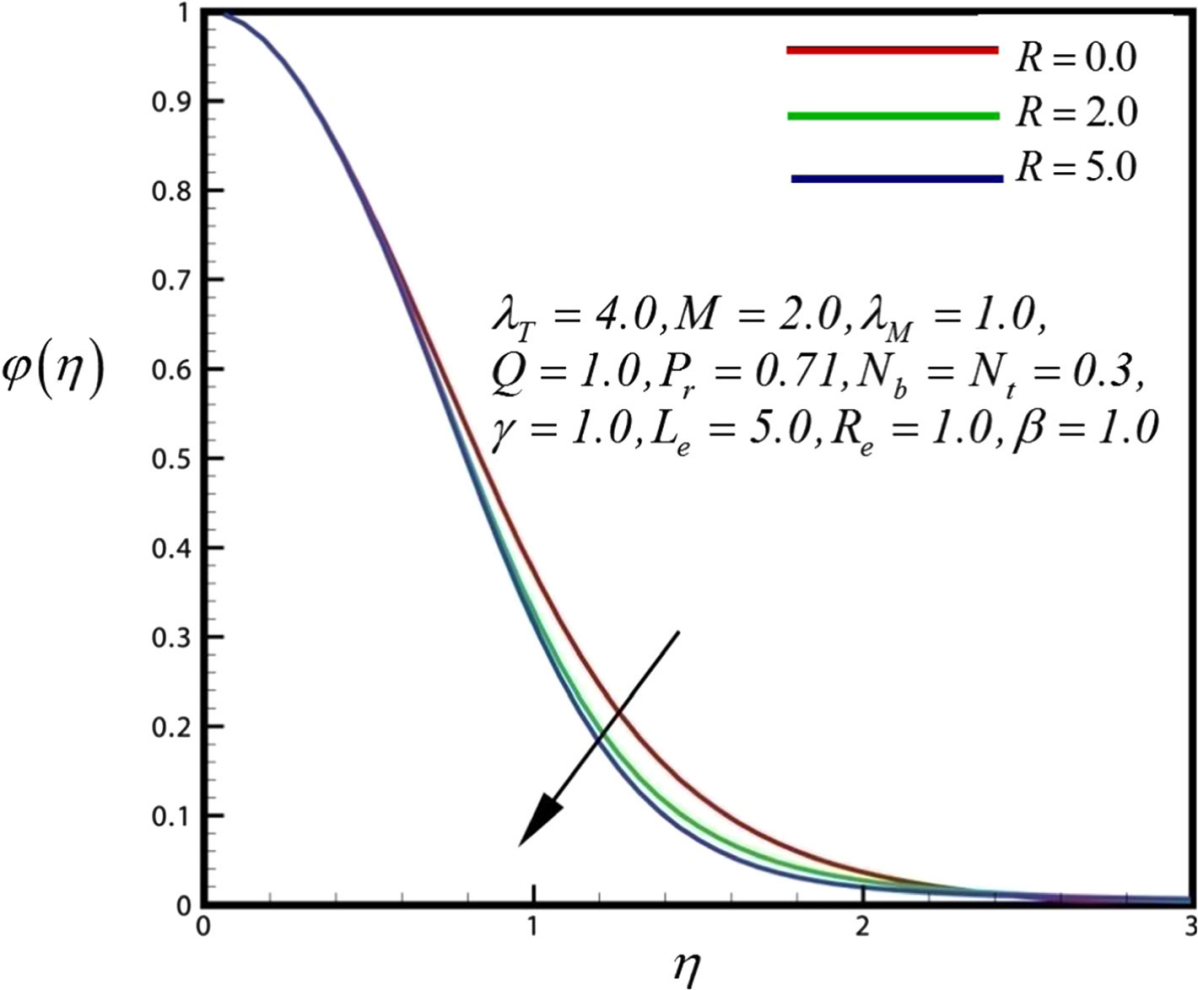
**Concentration profile for different values of** ***R*** **.**

Figure 2 exhibits the dimensionless velocity distribution for different values of thermal convective boundary parameter (*λ*_*T*_). The momentum boundary layer follows the boundary condition due to the shrinking wedge. An increase in thermal convective (thermal buoyancy) parameter is observed to strongly accelerate the flow. Therefore the velocity profiles are rises within the boundary layer as the *λ*_*T*_ increase. Figure 3 represents the dimensionless velocity distribution for different values of mass convective parameter (*λ*_*M*_). Near the shirking wedge surface it was found that, with increasing the mass convective (species buoyancy) parameter serves to increase the velocity profile. Therefore increasing species buoyancy force therefore only aids momentum and increases velocity boundary layer thickness further from the sheet. It was also observed that the momentum boundary layer follows the boundary condition due to the shrinking wedge. Figure 4 illustrate the dimensionless velocity distribution for different values of heat generation parameter (*Q*). Then for above case it is observed that velocity profiles are increases as the *Q* increase. It was also found that the shrinking wedge follows the boundary condition.

Figure 5 displays the velocity distribution for different values of radiation parameter (*R*). An increase in radiation parameter (*R*) is found to slightly upturn velocity values close to the sheet because of the flow is accelerated therefore momentum boundary layer thickness is therefore increased strongly after some distance from the wall. Also the shrinking wedge follows the boundary condition appropriately. With increasing thermal convective parameter as plotted in Figure 6, temperature distribution is increased significantly. Increasing thermal buoyancy force aids momentum development which results in a rise in temperature and a concomitant increase in thermal boundary layer thickness. Figure 7 represents the temperature distribution for different values of *λ*_*M*_. An increase in mass convective (species buoyancy) parameter, as shown in Figure 7, generally heats the boundary layer and causes temperatures to decrease in the nanofluid.

Close to the wedge surface, heat generation parameter (*Q*) (Figure 8) serves to slightly increase the temperature distribution, whereas further from the sheet the reverse behavior is observed and the flow is accelerated. Increasing heat generation therefore only aids temperature and increases temperature boundary layer thickness further from the wedge. Figure 9 displays the dimensionless temperature distribution for different values of *R*. The consequence is stable for all distances into the boundary layer and validates the advantage of employing thermal radiation in nano-scale-materials dispensation processes. Through growing radiation temperature in the nanofluid is significantly intensified. *R* represents the comparative contribution of thermal radiation heat transfer to thermal conduction heat transfer. Subsequently thermal radiation augments the thermal diffusivity of the nanofluid, for increasing values of *R* heat will be added to the regime and temperatures will be increased.

Figure 10 portrays the dimensionless concentration distribution for different values of *λ*_*T*_.A strong decrease in the nano-particle concentration is caused by increasing the thermal convective parameter has found. A slightly increase in the nano-particle concentration is caused by increasing the mass convective parameter (*λ*_*M*_) in Figure 11. Concentration boundary layer thickness is therefore higher with increasing species buoyancy force.

Figure 12 depicts the dimensionless concentration distribution for different values of *γ*. Nano-particle concentration and concentration boundary layer thickness is decreased with strong chemical reaction closer to the sheet surface, whereas the opposite trend is computed further from the sheet surface. Therefore finally concentration boundary layer thickness is increases. Figure 13 illustrates the dimensionless concentration distribution for different values of heat generation parameter. Nano-particle concentration and concentration boundary layer thickness is decreased with strong heat generation parameter closer to the sheet surface, finally concentration boundary layer thickness is increases further from the sheet surface. Figure 14 displays the dimensionless concentration distribution for different values of *R*. Through rising radiation (*R*), concentration in the nanofluid is significantly diminished.

## 5 Conclusions

In this study, the governing equations for the considered MHD radiative, heat-generating and chemical reacting nanofluid flow past a wedge is presented. A numerical representation has been developed for the boundary layer flow past a wedge with the influence of thermal radiation, heat generation and chemical reaction. It was found a good accuracy of skin friction coefficient which is compared with the previous studies [27,29-31].

The significant findings of present study are given below:

- The velocity profiles are rises within the boundary layer as the *λ*_*T*_, *λ*_*M*_, *Q* and *R* increase correspondingly.
- With increasing independently thermal convective parameter and radiation than temperature increased significantly. Whereas the opposite effect has been found for mass convective (species buoyancy) and heat generation parameter respectively.
- A strong decrease in the nano-particle concentration is caused by increasing the thermal convective parameter and radiation individually. But it rises for increasing mass convective, chemical reaction and heat generation parameter.

### 5.1 Nomenclature

*A*, positive constant

*B*_*o*_, magnetic induction

_*C*_, positive constant

*C*, nanoparticle concentration

*C*_*w*_, nanoparticle concentration at wedge

*C*_*∞*_, ambient nanoparticle concentration as y tends to infinity

*c*_*p*_, specific heat capacity

*D*_*B*_, Brownian diffusion coefficient

*D*_*T*_, thermophoresis diffusion coefficient

*G*, acceleration due to gravity

*G*_*r*_, Grashof number

*G*_*m*_, Modified Grashof number

*K*_*r*_, rate of chemical reaction

*L*_*e*_, Lewis number

*M*, positive constant

*M*, magnetic parameter

*N*_*b*_, Brownian motion parameter

*N*_*t*_, Thermophoresis parameter

*P*, fluid pressure

*P*_*r*_, Prandtl number

*Q*, heat source parameter

*Q*_*o*_, heat generation constant

*R*_*e*_, local Reynolds number

*T*, fluid temperature

*T*_*w*_, temperature at the wedge surface

*T*_*∞*_, ambient temperature as y tends to infinity

*x, y*, Cartesian coordinates measured along wedge

*u, v*, velocity components along *x* and *y* axes respectively

*Greek symbols*

*Ν*, dynamic and kinematic viscosities

*(ρc)*_*p*_, effective heat capacity of the nanofluid

*(ρc)*_*f*_, heat capacity of the fluid

*σ*, charge density

*α*, thermal diffusivity

*β*, pressure gradient parameter

*β*_*T*_, co-efficient of thermal expansion

*β*_*C*_, co-efficient of mass expansion

*λ*, constant moving parameter

*γ*, Chemical reaction parameter

*λ*_*T*_, thermal convective parameter

*λ*_*M*_, mass convective parameter

*η*, similarity variable

*ψ*, stream function

*f(η)*, dimensionless velocity

*θ(η)*, dimensionless temperature

*φ(η)*, dimensionless concentration
